# Assessing a Methodology for Evaluating the Latency of IPv6 with SCHC Compression in LoRaWAN Deployments

**DOI:** 10.3390/s23052407

**Published:** 2023-02-22

**Authors:** Emiliano Sisinni, Dhiego Fernandes Carvalho, Alessandro Depari, Paolo Bellagente, Alessandra Flammini, Marco Pasetti, Stefano Rinaldi, Paolo Ferrari

**Affiliations:** Department of Information Engineering, University of Brescia, Via Branze 38, 25123 Brescia, Italy

**Keywords:** LoRaWAN, SCHC, IoT

## Abstract

The Internet of Things (IoT) approach relies on the use of the Internet Protocol (IP) as a pervasive network protocol. IP acts as a “glue” for interconnecting end devices (on the field side) and end users, leveraging on very diverse lower-level and upper-level protocols. The need for scalability would suggest the adoption of IPv6, but the large overhead and payloads do not match with the constraints dictated by common wireless solutions. For this reason, compression strategies have been proposed to avoid redundant information in the IPv6 header and to provide fragmentation and reassembly of long messages. For example, the Static Context Header Compression (SCHC) protocol has been recently referenced by the LoRa Alliance as a standard IPv6 compression scheme for LoRaWAN-based applications. In this way, IoT end points can seamlessly share an end-to-end IP link. However, implementation details are out of the specifications’ scope. For this reason, formal test procedures for comparing solutions from different providers are important. In this paper, a test method for assessing architectural delays of real-world deployments of SCHC-over-LoRaWAN implementations is presented. The original proposal includes a mapping phase, for identifying information flows, and a subsequent evaluation phase, in which flows are timestamped and time-related metrics are computed. The proposed strategy has been tested in different use cases involving LoRaWAN backends deployed all around the world. The feasibility of the proposed approach has been tested by measuring the end-to-end latency of IPv6 data in sample use cases, obtaining a delay of less than 1 s. However, the main result is the demonstration that the suggested methodology permits a comparison of the behavior of IPv6 with SCHC-over-LoRaWAN, allowing the optimization of choices and parameters during deployment and commissioning of both infrastructure components and software.

## 1. Introduction

The advantages offered by the Internet of Things (IoT) paradigm are well known, as demonstrated by the ever-increasing number of applications based on the IoT in very diverse application scenarios [1]. One of the fundamental IoT pillars is the ubiquitous communication capability, to ensure the connectivity to many devices, possibly deployed over large areas. Wireless communications are often preferred, because of the flexibility and scalability they offer.

However, wireless performances are frequently traded with low-power consumption, as for the so-called Low Power Wide Area Network (LPWAN) technologies [2,3,4]. LPWAN is a family grouping different protocols offering a wireless tier for end-device connectivity, and another wired/wireless tier for backend and end-user integration. The preferred topology is the star, which ensures good efficiency and simplicity. Good coverage is obtained by leveraging on high sensitivity radios, with a relatively limited bandwidth and data rate. LPWAN technologies can use both licensed and unlicensed spectrum bands. Referring to the unlicensed bands, LoRaWAN has gained a large consensus in recent years [5,6,7]. Released in 2015, it immediately attracted interest from both industry and academia. The specifications, managed by the LoRa Alliance industrial consortium, define the protocol stack for the wireless tier, and the roles of logical entities in the backend. Both private and public backends can be realized, executed by on-premises servers, or offered as cloud services. Additionally, the end device is based on low-cost hardware provided by many manufacturers.

The architecture of an IoT application integrated with LPWAN normally includes edge devices, also known as gateways, whose job is to gather field devices’ messages by means of local connectivity, e.g., low power wireless, and to adapt the information in order to send it to the middle destination: the backend. The backend is in the cloud, it runs business intelligence and may store huge quantities of data. Last, the end applications, e.g., users or machines, access the backend by means of message oriented protocols, mostly in cases with a publisher/subscriber paradigm, and retrieve the needed data [8]. When the traffic is required to be bidirectional, the reverse path must be implemented. Notably, in order to assure scalability, devices at one end and applications at the other end cannot directly talk to each other, but they must pass through the backend.

The end user integration is typically with the cloud, where data are stored and processed, using an IP-based protocol. On the other hand, end-device integration is “opaquer,” backend-specific, and often non-IP-based. A great benefit for the full integration of IoT systems is the adoption of IP along the entire path of the IoT architecture. Among the advantages, the most important is the availability of well-known components/methods for IP management, IP security, and shared application profiles. Straightforward integration of IoT applications with other IP-based systems is also possible, such as the one addressed in [9]. For instance, this would improve IoT interoperability by means of web technologies [10] moving toward the scenario called Web of Things [11]. Additionally, availability of a global address can also help in forwarding messages in heterogeneous networks, as suggested in [12,13]. Unfortunately, IP overhead can be blocking for a certain part of IoT local connectivity technologies that only manage small frames.

For addressing this issue, the Internet Engineering Task Force (IETF) for LPWANs released the SCHC (Static Context Header Compression) [14] in 2020. SCHC enables the use of IP-based communications also on the end-device level by compressing IPv6 and UDP headers in order to fit the (usually) small payload of LPWAN networks. In detail, the SCHC standard works by compressing the static contexts of many IoT applications, where no relevant changes occur in the operation phase after an initial activation phase (which is usually carried out offline). In such a case, the header compression is based either on a priori known rules or on rules inferable from the information of other headers. For instance, considering LoRaWAN, which is currently the most diffused example of LPWAN [15], an additional standard for the use of SCHC has been available since 2021.

In the literature, both performance and feasibility of the SCHC have been investigated in several studies [16,17,18]. In [19], the wireless transmission airtime modification using SCHC has also been studied. In particular, the evaluation of latency (and of its variability, i.e., the jitter) is of main importance in any distributed system to decide if the communication infrastructure is suitable for the target applications. Indeed, supervision and control applications require low latency, while monitoring may tolerate higher latency. According to the authors’ best knowledge, no previous studies describing the methodology for evaluating end-to-end (round-trip) time performance in real-world SCHC-over-LoRaWAN deployments have been published. As a consequence, this present study is focused on filling this gap, providing usable methods and metrics for end users and IoT system owners.

The main contributions of this study are as follows:

- The introduction of a novel methodology for assessing the performance of IPv6 with SCHC-over-LoRaWAN;
- The definition of figure of merits, based on simple but effective metrics that can be mapped on different communication stacks, to allow a fair comparison of different deployments/implementations. Performance assessment can be exploited by the user and the manager of the IoT application to make decisions about the most suitable solution to adopt;
- The use of the proposed methodology to evaluate the performance in a realistic use case scenario with multiple implementations.

The paper is arranged as follows. In Section 2, a brief overview of LoRaWAN is provided. In Section 3, the SCHC is described. Then, the proposed methodology is introduced in Section 4. Section 5 discusses the considered use case. In Section 6, the experimental results are reported, and the effectiveness of the proposed methodology is shown. Finally, the conclusions are discussed in Section 7.

## 2. LoRaWAN Overview

In this section, an overview of main LoRaWAN characteristics is provided, followed by some additional notes about the use of bidirectional traffic.

### 2.1. The LoRaWAN Solution

LoRaWAN open standard specifications are managed by the LoRa Alliance consortium and describe the upper-level protocols of the communication stack lying above a radio physical layer (PHY). The PHY usually consists of LoRa radio, an enhanced chirp spread spectrum modulation capable of coding SF (the Spreading Factor, where SF ∊ [7, …, 12]) bits of information in a single upchirp [20]. Indeed, LoRa divides the upchirp duration T_C_ into 2^SF^ possible, equally spaced time instants; depending on the symbol to be transmitted, a discrete jump in the upchirp frequency trajectory is added, wrapping around the chirp bandwidth B. The bandwidth B is fixed (and depends on the regional regulation, e.g., in Europe B ∊ [125, 250] kHz), while the chirp duration obeys the relationship T_C_ = 2^SF^/B. A longer chirp length increases the resilience to interference and improves the sensitivity, enlarging the covered area, owing to the additional processing gain they permit. On the other hand, the data rate (exponentially) decreases. Another advantage of changing the SF is the quasi-orthogonality of time overlapping symbols transmitted using different SF values. In other words, SF can be considered as a type of virtual channel, thus counteracting the scarcity of radio resources for unlicensed spectrum regions. The medium access control (MAC) exploits the pure ALOHA strategy, while the network (NWK) topology mimics the “hub-and-spoke” approach of star networks [21,22].

**Figure 1:**
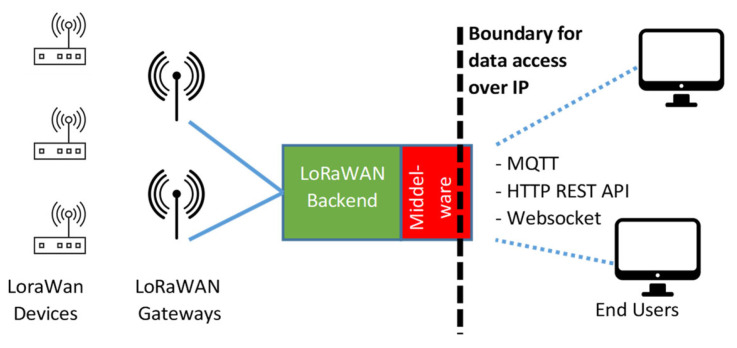
Block diagram of a regular LoRaWAN solution, where a wireless tier is connected to the wired tier (the backend) via GWs. In this case, the middleware providing integration with the end users marks the boundary where data are accessible over the IP protocol.

However, the most interesting feature of the LoRaWAN standard is the description of the backend, which consists of several logic entities, named “servers” [23]. Indeed, end devices are one hop away from gateways (GWs), which tunnel uplink (UL) messages from the field into the backend and do the opposite for the downlink (DL) messages. All the GWs in a LoRaWAN network are connected with the Network Server (NS), that actually manages the network behavior (e.g., exploiting the available data rates for implementing Adaptive Data Rate (ADR) strategies). The NS is connected to one or more Application Servers (ASs) that permit end user integration, usually by means of additional middleware [24]. A typical example is the adoption of the MQTT protocol, leveraging on a Broker for forwarding messages, identified by “topics”, from the publisher towards the subscribers. Other integration technologies may include HTTP-based approaches, such as REST API, Web Socket, and Web Hooks, etc. Therefore, in a typical LoRaWAN solution, the AS (and the adopted integration strategy) marks the boundary where data are accessible over the IP protocol, as shown in Figure 1.

### 2.2. Bidirectional Traffic

Most IoT applications can be classified as “monitoring” applications and, thus, only need to move data from the field towards the end user. Additionally, downlink traffic consumes resources to the detriment of uplink traffic [25]. However, bidirectionality occurs for a variety of reasons, which includes (i) the (over-the-air) activation procedure for new end devices; (ii) the (dynamic) management of the infrastructure (e.g., LoRaWAN defines MAC level commands, enabling link connectivity testing and parameter adaptation); (iii) acknowledging critical messages; and (iv) providing feedback from the end-user application.

Regarding LoRaWAN, the duty cycle limitation, depending on the region of operation, which is typically in the order of 1% per sub-band, is probably the most challenging. Moreover, radios in the GWs are half-duplex; when uplink frame reception is ongoing, scheduling of the downlink frame can be delayed. Indeed, while GWs are generally designed to handle multiple frames simultaneously, if transmitted via different channels and/or SFs, they are generally capable of transmitting only one downlink frame at a time. However, it is worth mentioning that both downlink and uplink exploit polarity inversion to minimize interferences (swapping the in-phase component of the baseband signal with the quadrature one). Consequently, frames from gateways are recognized only by end devices and vice versa, avoiding the problem of an ongoing node transmission interfering with a gateway concurrent one. However, being that the radio link is intrinsically half-duplex, increasing the downlink traffic lowers the opportunity to receive uplink traffic.

However, bidirectionality is also affected by the transmission pattern implemented by LoRaWAN devices. Nodes are grouped into three classes: A, B, and C [26]. Class A features are mandatory; communication is started by the end device (e.g., triggered by an event of interest) and determines the opening of two reception windows (RX1 and RX2) after a configurable time since the end of the uplink frame (T_RX1_ and T _RX2_ = T _RX1_ + 1 s). Consequently, feedback information from the end-user application must wait for an uplink communication to be effectively scheduled. In order to overcome this limitation, class B adds time synchronization (forcing GWs to periodically send special downlink Beacon frames) so that additional reception windows can be opened between successive transmissions. Finally, class C permits the shortest round-trip-time, since the RX2 window is extended up to the next uplink frame reception. Obviously, such a behavior greatly affects the power consumption and class C operations are generally enabled when mains power supply is available.

As a concluding remark, the usage of downlink messages must be carefully evaluated, since increasing their generation rate adversely affects the goodput of traffic in both directions, as already pointed out in the literature [27]. For instance, one very popular LoRaWAN backend, “The Things Network—Public Community”, has a fair use policy which limits the number of downlink messages to 10 messages per node per day.

## 3. The SCHC Compression Strategy

One of the main limitations preventing the usage of the IPv6 protocol in typical (wireless) IoT-like communication solutions is the reduced available payload, constrained by both the underlying radio technology and the need for power consumption minimization. This is the case for the LoRaWAN technology; the aforementioned LoRa radios offer a raw bit rate in the range Rb ∊ {0.293, …, 5.470} kbps when SF ∊ {12, …, 7}, CR = 4/5, and BW = 125 kHz, and they exploit the EU868 frequency plan available in Europe. If a fewer number of channels is tolerated, selecting BW = 250 kHz permits the data rate to be doubled (i.e., Rb ∊ {0.586, …, 10.940} bps). In any case, the available payload of a single frame depends on the actual data rate and, if all the SFs must be considered, specifications define a 51 B upper bound to ensure a reasonable airtime, even for slower transmissions. Therefore, the 40 B length of the fixed IPv6 header introduces a huge overhead, which is not tolerable even if payload compression is applied [28]. For this reason, header compression strategies have been proposed in the past to avoid redundant information and minimize the header size [28,29]. Header compression strategies are generally addressed as stateless, stateful, and hybrid. The first group includes all the solutions that do not require the sender and the receiver to preliminary exchange information about the way the header must be decompressed. On the contrary, the other two groups are more efficient (since they can dynamically adapt and modify the compression algorithm), but require the sender to share the context with the receiver.

Shortly after the advent of wireless sensor network based on IEEE802.15.4 radios, a stateless compression algorithm was formalized in the RFC4944 (the so-called 6LoWPAN [30,31,32]), allowing the IPv6 and the UDP headers to fit in a 2B- and 4B-long fields, respectively. Better performance is obtained following the strategy in the RFC5795 or in the RFC6282 [33], both implementing a stateful approach, that, in turn, results in wasting power and spectrum resources due to the additional context synchronization. In [34,35], efforts were made to port 6LoWPAN and the RPL routing protocol on LoRa, but not on LoRaWAN.

**Figure 2:**
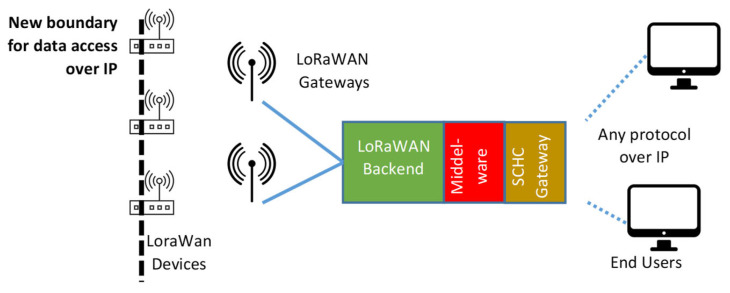
Block diagram of a SCHC-based LoRaWAN solution, where data are accessible over the IP protocol at the end-device level.

In order to minimize the compression cost as much as possible, the IETF’s LPWAN workgroup proposed the SCHC [19,36], formalized in the RFC8724, a static compression and fragmentation solution targeting the exchange of IPv6/UDP packets in LPWAN networks. In this way, both communication endpoints can be natively addressed using IPv6 addresses and transparently exploit typical upper-level protocols based on UDP (or even the TCP) transport mechanism, including security mechanisms [36,37,38]. A pictorial description for the SCHC-LoRaWAN case is provided in Figure 2, showing that the boundary for accessing data over the IP protocol is now moved into the end device (differently from Figure 1).

**Figure 3:**
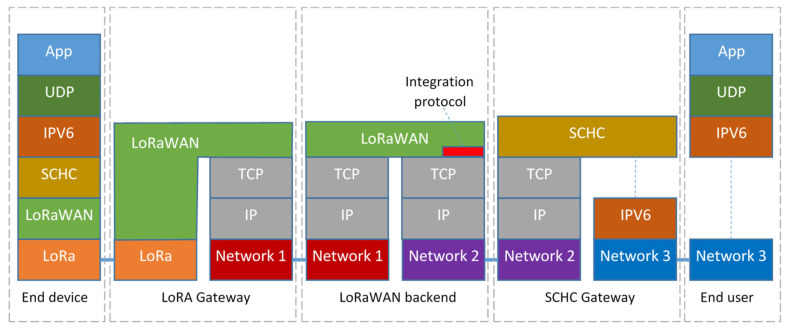
Example of encapsulation of the (TCP)UDP/IPv6 protocol in LoRaWAN networks via the SCHC adaptation layer.

The way the application data are (de)encapsulated in protocol stacks of the different devices involved in the message forwarding is shown in Figure 3.

The SCHC approach relies on the high predictability of the traffic generated in typical LPWAN applications. Therefore, both ends of the communication can exploit a pre-shared, static context that provides the header information. Such a context consists of a set of “rules” identified by a fixed size “Rule-ID”, that improves flexibility and performance permitting to dynamically change the compression configuration based on the actual traffic. Indeed, if traffic variability is low, changes in the header fields are small as well, and it is possible to a priori identify a set of well-known header configurations (the Rule-ID) stored at both the source and destination endpoints. As a consequence, the identifier of the rule closest to the actual uncompressed header is transmitted, complemented by a possible “residue” field for all the information that cannot be retrieved from the Rule-ID alone. Obviously, when the residue is empty, the shortest message length can be obtained.

**Figure 4:**
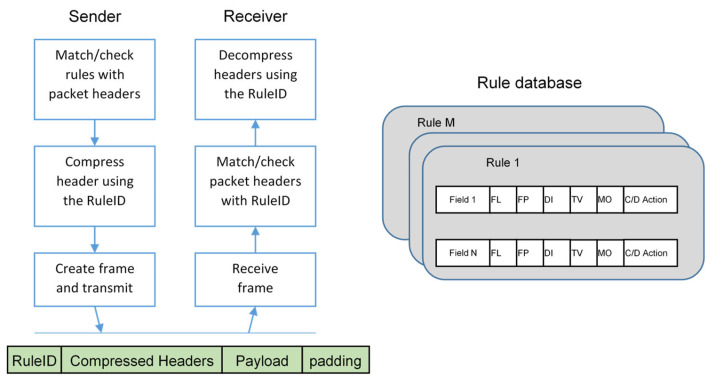
Compression and decompression steps according to the rules that describe the context in the SCHC approach.

A single rule includes several fields, derived from the original header parameter, which are (i) the Field ID (FID): an identifier univocally representing the header field; (ii) the Field Length (FL), representing the length of the header segment; (iii) the Field Position (FP): used for selecting an individual item when the field is an array; (iv) Direction Indicator (DI): distinguishing between uplink, downlink or bidirectional traffic (i.e., from the field to the backend, the opposite or both, respectively); (v) the Target Value (TV) and the Matching Operator (MO): representing the value (TV) and the operator (MO) for matching with the original header field; (vi) the Compression Decompression Action (CDA): specifying the method used for actually compressing and decompressing the TV value. A simplified graphical description is provided in Figure 4.

**Figure 5:**
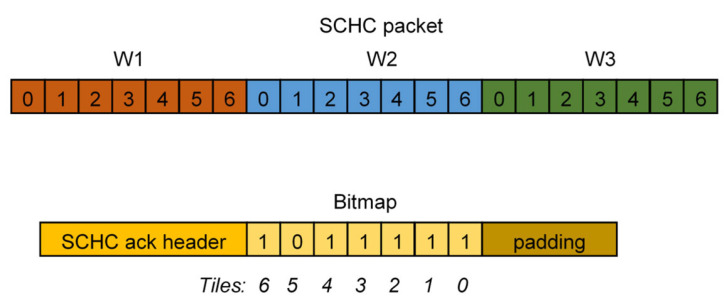
Example of an SCHC packet with three windows W of seven tiles each and the bitmap in the SCHC ACK describing if there are missing tiles in the acknowledged window (note that tile 5 is missing in this case.).

However, minimizing the header overhead does not allow handling the large Maximum Transmission Unit (MTU) that characterizes IPv6 traffic. Moreover, most LPWAN technologies do not provide a native solution for slicing the original application payload into a set of smaller fragments [39]. For this reason, the SCHC also defines how the packets are disassembled and reassembled. The RFC8724 specifies that each fragment consists of several disjoint “tiles”, grouped into different “windows”; if reunited, the original packet is obtained. The tile and window cardinality are both configurable parameters and fragments are individually addressed via the pair {tile index and window number}. Therefore, fragments consist of a header followed by a payload that can contain tiles related to the original IPv6 header and/or payload. The fragment header carries the Rule-ID, the window size, the fragment number, and an integrity check sequence. Different reliability modes are defined, trading efforts in error detection with latency: No ACK, ACK Always, and ACK on Error. The first solution can be considered a best effort delivery strategy, since no feedback about fragment reception is provided. On the contrary, the remaining two strategies exploit the reception of an ACK for selectively retransmitting lost fragments. Indeed, transmissions are acknowledged and, in case the integrity check fails, a bitmap describes which tiles have not been correctly received (see Figure 5). This bitmap is always sent in the ACK Always mode. On the contrary, if ACK on Error is enabled, the bitmap is sent only if some tiles are lost. As a result, ACK loss can be very expensive in terms of resource cost in the latter case, and an additional final ACK, reporting all the missing “tiles”, can also be sent to minimize the overall impact.

It must be highlighted that, since May 2022, the LoRa Alliance integrates the LoRaWAN specifications to officially support the SCHC compression scheme targeting additional use cases in smart metering, Industry 4.0, smart buildings, smart homes, etc. When the LoRaWAN LPWAN is considered, the aforementioned RFC9011 is applied and the RuleID, which is 1B long, is transmitted using the “Fport” field of the LoRaWAN header. Additionally, coding uplink and downlink frameswith the RuleID #20 and #21 is also suggested.

## 4. The Proposed Methodology

Objective and reproducible performance assessment of communication networks requires a well-defined methodology [40]. As pointed out in a recent paper [15], relevant studies discussing models and strategies for evaluating different aspects of the SCHC performance applied to LPWAN technologies (namely, Sigfox and LoRaWAN) are ongoing, but uncertainty about the actual obtainable performance remains. In particular, it highlights that only one paper [18] presents experimental results for assessing SCHC-over-LoRaWAN performance. Moreover, according to the authors’ best knowledge (i) there is no literature about the end-to-end latency when the backend is also included in real-world deployment, and (ii) a formal, generally applicable approach for metrics estimation that can be easily replicated by other researchers has not been provided.

The aim of this work is to fill in this gap, providing a methodology for estimating the time performance of a bidirectional data exchange based on the UDP/IPv6 protocol when the SCHC compression strategy is adopted over a real-world LoRaWAN deployment. In particular, it is expected that a field device (i.e., an end node) starts event-based communication towards a remote user application, that subsequently replies with a feedback message. As typically occurs in IoT applications, the backend is hosted in the cloud and integration of end devices and users occurs via a type of message-oriented middleware/protocol.

The vast literature available confirmed that the LoRaWAN, using a very simple medium access strategy (i.e., a type of pure ALOHA exploiting a limited number of channels and a reduced bandwidth and duty-cycle), suffers from poor end-to-end performance in critical scenarios, when a high number of co-located devices must exchange data at a high update rate [41]. However, few studies have characterized the backend, where most of the computational and storage efforts are carried out and, as previously stated, there are no studies where the SCHC is included. Moreover, the specifications only provide a loose description of the logical entities in the backend (the NS, AS, and JS servers), so that very different setups can be implemented. As a consequence, performance evaluation and comparison in real-world deployments can be a non-trivial task. When the SCHC is considered, a further adaption layer is present, further increasing the setup complexity.

### 4.1. The Proposed Methodology: Goals

The aim of the proposed methodology is to provide a general approach for evaluating not only the wireless tier, consisting of the wireless star network linking nodes and the closest gateway, but also the backend tier in the presence of SCHC. In detail, the backend entities are abstracted into “black boxes”, according to the LoRaWAN specification description, and the attention is focused on the information flow rather than on peculiar details of the actual implementation. Moreover, no architecture changes and/or additional code probes are required. Since bidirectional data exchanges are considered, both uplink and downlink data flows must be characterized [42].

Regarding the SCHC implementation, header compression and decompression must be performed on the end-device side (i.e., in the mote on the field) and on the user application side, after data are retrieved from the LoRaWAN backend for the uplink direction, or before data are provided to the LoRaWAN backend for the uplink direction: “SCHC gateways” must be present on both ends of the communication.

**Figure 6:**
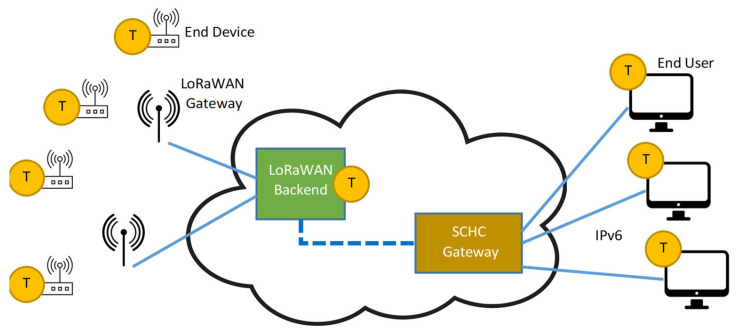
Proposed methodology: general overview of a typical LoRaWAN-SCHC deployment, showing timestamping points for metrics evaluation.

The end-device traffic is injected into the infrastructure under test by means of a probing node and it is gathered by the end-user application via the SCHC gateway. Similarly, the end-user traffic is injected via the SCHC gateway, upon reception of the uplink data, in order to reach the probing node. In order to quantify the delays, time information must be provided; well-defined timestamping points must be specified, depending on the actual implementation. A general overview of a typical LoRaWAN-SCHC deployment, showing timestamping points “T” for metrics evaluation, is provided in Figure 6. For simplicity, the NS, the AS, and the (optional) JS are hidden in the “LoRaWAN Backend” block.

**Figure 7:**
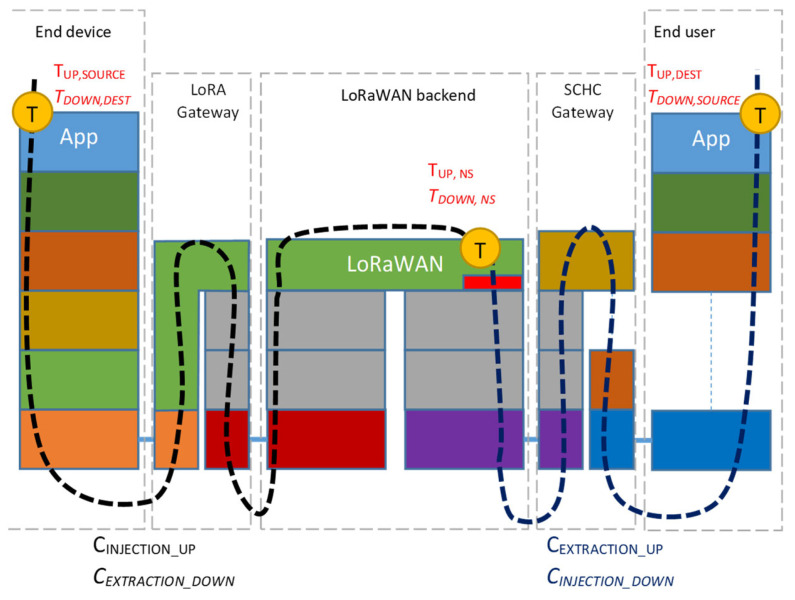
Proposed methodology: identification of data flows for uplink and downlink directions *(italics*); identification of timestamping points (yellow dots); and identification of timestamps for uplink and downlink (*italics*).

The actual data flow is sketched in Figure 7. Regarding the uplink direction, it can be split into the following sub-components:

- C_INJECTION_UP_: from the end-device to the NS (data injection into the LoRaWAN network); it includes the wireless part of the overall uplink data flow;
- C_EXTRACTION_UP_: from the NS to the end-user application (data extraction from the LoRaWAN network); it includes data forwarding via the Internet.

Similarly, for the downlink direction, the sub-components are as follows:

- C_INJECTION_DOWN_: from the end-user application towards the NS (data injection into the LoRaWAN network);
- C_EXTRACTION_DOWN_: from the NS to the end-device mote (data extraction from the LoRaWAN network).

Without losing generality, timestamping points “T” can be collected by (i) the end device; (ii) the end-user applications; and (iii) the backend (typically when the message reaches the NS).

### 4.2. The Proposed Methodology: Steps

As previously stated, the proposed methodology consists of the following steps:

(1) Identification and block mapping (first preparation step): devices and software used in the actual deployment must be matched against the abstract entities previously described, so that boundaries among them can be identified and information ingress and egress ports can be highlighted. In general, the uplink direction can be split into N flows F_UPx_, x = 0 … N−1, while M flows can be identified for the downlink direction F_DOWNy_, y = 0 … M−1.
(2) Timestamping point configuration (second preparation step): each identified data flow sub-component defines up to two timestamping points (providing origin and arrival timestamps). In general, each uplink flow F_UPx_ is marked by a flow ingress timestamp T_UPx,i_ and a flow egress timestamp T_UPx,e_. Similarly, for flow F_DOWNy_, timestamps T_DOWNy,i_ and T_DOWNy,e_ mark the ingress and egress instants for the downlink direction. Time synchronization is needed to ensure a common sense of time among all the actors and physical adaptation can be also required depending on the way the desired functionality is implemented.
(3) Measurements and analysis (execution step): the infrastructure under test is stimulated, and the information flows in the uplink and downlink directions are monitored and measured. The performance analysis is carried out by evaluating the differences between the timestamps gathered for the same data flow. In general, uplink traverse flow delay can be evaluated as L_UPx_ = T_UPx,e_ − T_UPx,i_. Similarly, downlink flow traverse latency can be evaluated as L_DOWNy_ = T_DOWNy,e_ − T_DOWNy,i_. Several different metrics can be defined, in order to highlight delays through architecture and internetworking blocks.

## 5. The Reference Real-World Deployment

**Figure 8:**
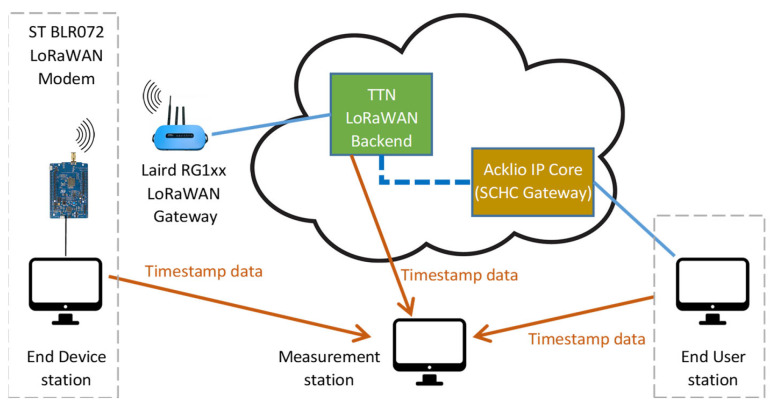
Setup of the reference real-world use case.

The proposed methodology has been tested in a real-world deployment, depicted in Figure 8, where the “Measurement station” used in the experiments is also shown.

The test application executed by the on-the-field mote is a simple Python program that periodically generates a 16B-long message, which is sent via UDP/IPv6 towards the test user (loopback) applications. The latter waits for the uplink data reception and replies, generating a message of the same length (i.e., 16 B), which is also sent via UDP/IPv6 towards the mote. The test application provides the following:

- T_UP,SOURCE_: timestamp (at the application level) of uplink data sending;
- T_DOWN,DEST_: timestamp (at the application level) of downlink data reception.

The test-user application is another Python-based program that provides:

- T_UP,DEST_: timestamp (at the application level) of uplink data reception;
- T_DOWN,SOURCE_: timestamp (at the application level) of downlink data sending.

The mote consists of a host station (a desktop PC based on Intel i7-8770, with 16 GB of RAM and running Debian Linux v11, kernel 5.10) connected with a LoRaWAN modem designed around the B-L072Z-LRWAN1 development kit from STM and running the Acklio Library. In this way, the LoRaWAN modem can be controlled by means of AT-like commands via a USB virtual serial link. The test end device was located in the Department of Control and Automation Engineering at the State University of São Paulo. Very close to the mote, the GW (an RG1XX from Laird) was installed; the good link quality ensures that no messages are lost on the wireless link due to weak packets and/or interferences with other intentional/unintentional radiators. The (loopback) test-user application is executed by the same desktop PC. Both the GW and the PC are connected to the Internet via the University broadband link.

Regarding the backend, nowadays, several open-source solutions exist, which are generally compliant with LoRaWAN specifications. In this work, the ChirpStack is considered, since it provides both the NS and AS that are ready to use, a user-friendly web-interface for management, and several integration methodologies. In particular, the backend was deployed in the cloud using Google Cloud; a Linux-based virtual machine (running Debian 11 distribution) was installed on a cost-optimized *E2-medium* machine based on Intel Broadwell processors with 2 vCPUs and 4 GB of memory. Different zones were selected in order to highlight the dependence from the backend location: asia_east_a (Hong Kong), eu_west8_a (Milan), and us-east1-b (South Carolina).

Regarding the SCHC implementation, the company Acklio recently released a complete solution for LoRaWAN that includes (i) the Acklio Library for developing the end-device application, abstracting the SCHC adaptation layer and lower levels of the protocol stack on the mote side; (ii) the Acklio IP Core, retrieving information from a LoRaWAN backend (natively supporting the ChirpStack) and providing integration with the end-user application. In this work, the authors selected the Acklio solution due to its compliance with the standards, thus avoiding relying on a private, purpose-designed adaptation layer.

The Acklio IP Core is connected via a VPN (Virtual Private Network) connection with test-user applications and accesses the LoRaWAN backend via a HTTPS protocol, so that the LoRaWAN backend forwards events of interest to the Acklio IP Core endpoint as POST requests. Since bidirectional data exchanges are in place, the end device behaves as a class C node operating in the EU868 frequency plan. ChirpStack integration allows information about uplink and downlink frames to be obtained, accessing “events” named “up” and “txack”, respectively. Timestamps are available, specifying when the event was actually published:

- T_UP,NS_: uplink frame reception by the NS;
- T_DOWN,NS_: confirmation sent by the NS that the downlink frame has been transmitted.

Following the approach detailed in [42], it was possible to evaluate the traversal time metrics L_UP_ and L_DOWN_, useful for comparing obtainable time performance, defined as follows:

- L_UP_ = T_UP,DEST_ − T_UP,SOURCE_, i.e., the end-to-end delay in the uplink direction;
- L_DOWN_ = T_DOWN,DEST_ − T_DOWN,SOURCE_, i.e., the end-to-end delay in the downlink direction;

Additionally, other insights about the delay sources can be obtained analyzing the other following metrics:

- L_UP,LoRa_ = T_UP,NS_ − T_UP,SOURCE_: the time spent to access the LoRaWAN backend (including the airtime across the wireless LoRaWAN link), for the uplink frame;
- L_UP,SCHC_ = T_UP,DEST_ − T_UP,NS_: the time needed for moving the uplink message from the NS to the end-user application (including the time spent in the Acklio IP Core);
- L_DOWN,SCHC_ = T_DOWN,NS_ − T_DOWN,SOURCE_: the time needed for moving the downlink message from the end-user application to the NS (including the time spent in the Acklio IP Core);
- L_DOWN,LoRa_ = T_DOWN,DEST_ − T_DOWN,NS_: the time spent in exiting the LoRaWAN backend (including the airtime across the wireless LoRaWAN link), for the downlink frame.

The metrics are actually computed by the “Measurement station” after gathering timestamp data from the end-device station, the end-user station, and the LoRaWAN backend.

## 6. Experimental Results

In this section, the results obtained using the proposed methodology on the real-world deployment are reported. Since the estimation of the previously defined metrics requires the collection of timestamps on machines located all around the world, time synchronization is needed.

### 6.1. Synchronization and Repeatability of Peers

In this work, the end-device application and the end-user (loopback) application were both executed by the same machine connected to the Internet via the broadband infrastructure of the State University of São Paulo; synchronization was obtained via the a.st1.ntp.br NTP (Network Time Protocol) server. Due to the good latency performance of this Internet link, synchronization uncertainty of millisecond order was expected. In particular, the accuracy was evaluated by analyzing the contributions of the synchronization and timestamping strategies; the contribution due to the hardware clock drift was ignored, due to the relatively short data exchange duration (on the order of a few seconds) and the good stability of the crystal-based oscillator. Regarding the synchronization accuracy, it was derived from the residual time offset RO (after the operating system clock was dynamically corrected, depending on the previous offset and delay measurements with respect to the NTP server), listed in the logs of the NTP client (chrony). Therefore, the experimental synchronization standard uncertainty *u_s_* was estimated as $u_{s}^{}=\sqrt{\mu_{RO}^{2}+\sigma_{RO}^{2}}$, where *μ_RO_* and *σ_RO_* are the average (uncompensated systematic component) and standard deviation values of the residual time offset, respectively.

The timestamping contribution *u_tn_* was evaluated executing a simple experiment; a Python script timestamps the instants immediately before (T_START_) and after (T_STOP_) the execution of a 1 s long delay function. Both T_START_ and T_STOP_ timestamps and the (software-depending) delay are based on the system clock of the same machine; therefore, Δ = (T_START_ − T_STOP_) − 1 s ≠ 0 because of the timestamp uncertainty *u_t_*. In particular, experimental uncertainty can be estimated as $u_{t}^{}=\sqrt{\frac{\mu_{\Delta }^{2}+\sigma_{\Delta }^{2}}{2}}$, where *μ*_Δ_ and *σ*_Δ_ are the average (uncompensated systematic component) and standard deviation values of the Δ interval, respectively. Experimental values are reported in Table 1.

Further, the standard uncertainty *u_xy_* of any metrics calculated as the time difference of y and x timestamping points can be modeled as $u_{xy}^{2}=u_{sx}^{2}+u_{tx}^{2}+u_{sy}^{2}+u_{ty}^{2}$. Analyzing the results in Table 1, it can be highlighted that, for cloud-based machines, the timestamp uncertainty dominates, whereas for local machines, the synchronization uncertainty is the dominant.

### 6.2. Use Case Results

An experiment was set up to demonstrate that the previously defined metrics can be used to evaluate the selected real-world use case.

In detail, a 16B user payload is generated by the end-device application in the local host machine (connected with the LoRaWAN modem), every T = 60 s. The modem, running the Acklio Code library encapsulates the payload into an UDP/IPv6 message and performs the SCHC compression, so that an unconfirmed uplink message with a maximum data rate (i.e., SF = 7) is sent. The resulting overall airtime is about 66.8 ms. Once received by the end-user application (via the LoRaWAN backend and the Acklio IP Core), a new UDP/IPv6 message is generated and sent back (again, via the Acklio IP Core and the LoRaWAN backend), generating a downlink frame, transmitted using SF = 12, since the node is configured as a class C device (for testing the best obtainable performance for bidirectional traffic), and the extended RX2 is adopted for the scheduling. The resulting airtime is about 1646.6 ms.

The above-described experiment was repeated three times, changing the backend location in the cloud. Each run last includes messages sent over a period of about 2 h (i.e., more than 120 samples are considered). During the experiments, all the transmitted messages completed the roundtrip. The obtained results are reported in Table 2, Table 3 and Table 4, depending on the LoRaWAN backend location. Comments about the experimental results are provided in the next Section 6.3.

### 6.3. Comments about Experimental Results

It is essential to highlight that the goal of this paper is to propose and demonstrate a coherent and clear methodology to evaluate the time performance of IPV6 applications over LPWAN, and LoRaWAN in particular. Since the feasibility of the measurements and their low uncertainty have been demonstrated in the previous sections, the following comments will focus on the further help that the methodology provides in retrieving meaningful insights into the considered use case. On the contrary, the performance of the use case components and backend are not important for the scope of the paper.

In particular, some issues arise from the analysis of the results reported in Table 2, Table 3 and Table 4. The first question that the proposed methodology highlights is the long time L_DOWN_ needed for the downlink direction compared to the uplink direction. The explanation of this long delay is in the wireless link, and it is due to the scheduling of LoRaWAN downlink message in the second window (RX2). In the current configuration parameter of the ChirpStack backend, the RX2 transmission takes place using LoRa SF12, which has the lowest data rate. If the user is not satisfied with the current result, they could modify that parameter to adapt/adjust the T_DOWN_ and then use the proposed methodology again to assess the new delays.

The second question that the proposed methodology reveals is the high variability (standard deviation) of L_DOWN_, but it is also clearly exposed that all this irregular behavior is due to the downlink scheduling L_DOWN,SCHC_. The explanation of this high variability is again in the current configuration parameter of the ChirpStack backend. The stack examines and schedules downlinks with a cyclic operation with period of 1 s. Since the internal cyclic operation of the stack is not synchronized with the incoming uplink, a random delay uniformly distributed between 0 and 1 s is expected. The proposed methodology can be applied again if the user decides to modify the cycle time of the stack to test the effects of the adjustments.

The third question that the proposed methodology shows is the link between the location of the cloud server running the backend and the T_UP_ and T_DOWN_. Even if it is expected that the location of servers may impact delays, the proposed methodology could help users to exactly quantify the reaction time of their systems. From the obtained results, there are very few differences between placing the backend in the US or in Europe, if the device and the end application are in Brazil. On the contrary, using a server in Asia could add 200 ms to both L_UP_ and L_DOWN_. Since IoT applications are usually globally deployed and managed, the power of the proposed methodology in this case is to facilitate the user to measure, compare, and decide what is best for this application.

## 7. Conclusions

The Internet Protocol (IP) is a fundamental pillar of the Internet of Things (IoT) paradigm. The IP acts as a collector for the very different upper-level and lower-level protocols adopted by end points. However, the requirements, in terms of scalability and flexibility, would require the adoption of the IPv6 version, characterized by a large overhead and huge maximum payload length. Despite these characteristics not being challenging (if not an advantage) in wired infrastructures, they are hardly tolerable in wireless IoT-like protocols tailored for implementation with limited resources. Therefore, the SCHC protocol was proposed as an adaptation level for compressing the header and managing the fragmentation of IPv6 (and UDP) messages. SCHC targets Low Power Wide Area networks and has been officially considered by the LoRa Alliance, managing the LoRaWAN specifications. Adopting SCHC enables a full end-to-end IP link among end nodes (smart things) and end-user applications.

In this work, a methodology for characterizing the time performance of real-world deployments of the LoRaWAN/SCHC solution was proposed, complementing existing studies based on simulations. The feasibility of the proposed methodology was tested, addressing the SCHC solution from Acklio, which was characterized using the set of proposed metrics. The results of this specific use case for a class C LoRaWAN node, leveraging on cloud-based backends located all around the world, showed that IPv6 end-to-end latency of a 16B-payload for the uplink and downlink directions is less than 1 s and 4 s, respectively. Moreover, the effectiveness of the proposed methodology was highlighted owing to the insights it can provide to the user (or manager) of the IoT application based on IPv6 over LoRaWAN. In particular, it was possible to clearly pinpoint (and then track) the backend configuration parameters that can be modified/optimized to match user desiderata.

## Figures and Tables

**Table 1:** NTP synchronization and timestamp uncertainty.

[ms]	*u_s_*	*μ_s_*	*σ_s_*	*u_t_*	*μ_t_*	*σ_t_*
Local	**1.56**	0.48	1.49	**0.93**	1.22	0.49
Cloud (ASIA)	**0.02**	0.02	0.01	**3.06**	3.05	0.12
Cloud (EU)	**0.01**	0.01	0.01	**3.10**	3.10	0.13
Cloud (USA)	**0.01**	0.01	0.01	**3.09**	3.09	0.13

**Table 2:** Statics of metrics evaluated when the LoRaWAN backend is located in Hong Kong.

Statistic	L_UP,LoRa_	L_UP,SCHC_	L_UP_	L_DOWN,SCHC_	L_DOWN, LoRa_	L_DOWN_
Mean [s]	0.537	0.268	0.805	1.237	2.585	3.804
Dev. Std. [s]	0.016	0.006	0.016	0.464	0.002	0.435
Skewness	4.64	5.38	4.20	1.94	−0.87	1.72
Max [s]	0.641	0.315	0.908	3.453	2.589	6.029
Min [s]	0.520	0.262	0.787	0.565	2.576	3.153

**Table 3:** Statics of metrics evaluated when the LoRaWAN backend is located in Milan.

Statistic	L_UP,LoRa_	L_UP,SCHC_	L_UP_	L_DOWN,SCHC_	L_DOWN, LoRa_	L_DOWN_
Mean [s]	0.496	0.141	0.637	0.982	2.625	3.597
Dev. Std. [s]	0.020	0.003	0.020	0.387	0.002	0.354
Skewness	7.27	2.14	7.09	1.63	−0.51	1.64
Max [s]	0.679	0.159	0.819	2.905	2.629	5.528
Min [s]	0.479	0.136	0.622	0.436	2.617	3.061

**Table 4:** Statics of metrics evaluated when the LoRaWAN backend is located in South Carolina.

Statistic	L_UP,LoRa_	L_UP,SCHC_	L_UP_	L_DOWN,SCHC_	L_DOWN, LoRa_	L_DOWN_
Mean [s]	0.436	0.198	0.634	0.961	2.687	3.651
Dev. Std. [s]	0.015	0.024	0.028	0.328	0.002	0.331
Skewness	4.78	10.27	7.12	0.38	−0.47	0.37
Max [s]	0.536	0.452	0.883	2.191	2.692	4.876
Min [s]	0.418	0.188	0.611	0.330	2.680	3.014

## Data Availability

Not applicable.
